# Transcriptome analysis of sex-biased gene expression in the spotted-wing *Drosophila*, *Drosophila suzukii* (Matsumura)

**DOI:** 10.1093/g3journal/jkac127

**Published:** 2022-05-19

**Authors:** Dan Deng, Shisi Xing, Xuxiang Liu, Qinge Ji, Zongzhao Zhai, Wei Peng

**Affiliations:** Hunan Provincial Key Laboratory of Animal Intestinal Function and Regulation, State Key Laboratory of Developmental Biology of Freshwater Fish, Hunan International Joint Laboratory of Animal Intestinal Ecology and Health, Hunan Normal University, Changsha 410081, China; Hunan Provincial Key Laboratory of Animal Intestinal Function and Regulation, State Key Laboratory of Developmental Biology of Freshwater Fish, Hunan International Joint Laboratory of Animal Intestinal Ecology and Health, Hunan Normal University, Changsha 410081, China; State Key Laboratory of Ecological Pest Control for Fujian and Taiwan Crops, Key Lab of Biopesticide and Chemical Biology, Ministry of Education, Institute of Biological Control, Fujian Agriculture and Forestry University, Fuzhou 350002, China; State Key Laboratory of Ecological Pest Control for Fujian and Taiwan Crops, Key Lab of Biopesticide and Chemical Biology, Ministry of Education, Institute of Biological Control, Fujian Agriculture and Forestry University, Fuzhou 350002, China; Hunan Provincial Key Laboratory of Animal Intestinal Function and Regulation, State Key Laboratory of Developmental Biology of Freshwater Fish, Hunan International Joint Laboratory of Animal Intestinal Ecology and Health, Hunan Normal University, Changsha 410081, China; Hunan Provincial Key Laboratory of Animal Intestinal Function and Regulation, State Key Laboratory of Developmental Biology of Freshwater Fish, Hunan International Joint Laboratory of Animal Intestinal Ecology and Health, Hunan Normal University, Changsha 410081, China

**Keywords:** sexual dimorphism, sex determination, olfactory, immunity, gene expression

## Abstract

Sexual dimorphism occurs widely throughout insects and has profound influences on evolutionary path. Sex-biased genes are considered to account for most of phenotypic differences between sexes. In order to explore the sex-biased genes potentially associated with sexual dimorphism and sexual development in *Drosophila suzukii*, a major devastating and invasive crop pest, we conducted whole-organism transcriptome profiling and sex-biased gene expression analysis on adults of both sexes. We identified transcripts of genes involved in several sex-specific physiological and functional processes, including transcripts involved in sex determination, reproduction, olfaction, and innate immune signals. A total of 11,360 differentially expressed genes were identified in the comparison, and 1,957 differentially expressed genes were female-biased and 4,231 differentially expressed genes were male-biased. The pathway predominantly enriched for differentially expressed genes was related to spliceosome, which might reflect the differences in the alternative splicing mechanism between males and females. Twenty-two sex determination and 16 sex-related reproduction genes were identified, and expression pattern analysis revealed that the majority of genes were differentially expressed between sexes. Additionally, the differences in sex-specific olfactory and immune processes were analyzed and the sex-biased expression of these genes may play important roles in pheromone and odor detection, and immune response. As a valuable dataset, our sex-specific transcriptomic data can significantly contribute to the fundamental elucidation of the molecular mechanisms of sexual dimorphism in fruit flies, and may provide candidate genes potentially useful for the development of genetic sexing strains, an important tool for sterile insect technique applications against this economically important species.

## Introduction

Sexual dimorphism in morphological, physiological, and behavioral characteristics is pervasive in animals. Females and males can differ diversely in body size, brain anatomy, symbiotic microorganisms, and immune responses (Fish 2008; Cachero *et al.* 2010; Markle *et al.* 2013; Rideout *et al.* 2015). Despite only slight or total absence of differences at the genomic level between females and males, the sex-biased expression occurs in different stages of embryonic, juvenile, and adult development and converges on the formation of sexual dimorphism phenotypic traits (Ellegren and Parsch 2007; Williams and Carroll 2009; Grath and Parsch 2016). Recently, using high-throughput sequencing techniques, sex-biased gene expression has been extensively explored in species of *Drosophila*, including *Drosophila* *melanogaster*, *Drosophila* *simulans*, *Drosophila* *yakuba*, and *Drosophila* *ananassae* (Swanson *et al.* 2001; Meiklejohn *et al.* 2003; Ranz *et al.* 2003; Zhang *et al.* 2004; Proschel *et al.* 2006; Haerty *et al.* 2007; Zhang *et al.* 2007; Jiang and Machado 2009; Assis *et al.* 2012; Grath and Parsch 2012; Khodursky *et al.* 2020), species of mosquitoes, *Aedes aegypti*, *Anopheles gambiae*, *Anopheles* *arabiensis*, *Anopheles* *minimus*, and *Anopheles* *albimanus* (Papa *et al.* 2017; Whittle and Extavour 2017), species of fruit flies, *Anastrepha fraterculus* and *Anastrepha* *obliqua* (Congrains *et al.* 2018), species of fire ants, *Solenopsis invicta* and *Solenopsis* *richteri* (Ometto *et al.* 2011), species of parasitoid wasps, *Nasonia vitripennis* and *Nasonia* *giraulti* (Wang *et al.* 2015), the stick insect *Timema californicum* (Djordjevic *et al.* 2021), the damselfly *Ischnura elegans* (Chauhan *et al.* 2016), and the cricket *Gryllus bimaculatus* (Whittle *et al.* 2020). These studies have repeatedly shown that male-biased genes evolved rapidly in *Drosophila* due to adaptive changes in amino acids resulting from sexual selection pressures concerning male–male and sperm competition (Swanson *et al.* 2001; Zhang *et al.* 2004; Proschel *et al.* 2006), while an opposite pattern occurred in mosquitoes with rapid evolution of female-biased genes due to their mating biology in which female–female competition for suitable males takes place (Papa *et al.* 2017; Whittle and Extavour 2017).

In *Drosophila*, sex chromosome constitution (XX or XY) leads to sexually dimorphic expression of *Sex lethal* (*Sxl*), the binary switch gene in regulation of both sexual development and dosage compensation (Erickson and Quintero 2007; Vicoso and Bachtrog 2013; Lucchesi and Kuroda 2015). Two X chromosome copies initiate the expression of *Sxl* gene, which directs female-specific splicing of downstream target gene *transformer* (*tra*) pre-mRNA, leading to functional Tra protein only in females. Tra protein interacts with Transformer-2 protein to bind the pre-mRNA of target genes *doublesex* (*dsx*) and *fruitless* (*fru*), which produce Dsx^F^ protein and a nonfunctional Fru protein in females. In the absence of *Sxl* expression due to one X chromosome copy in males, male-specific *tra* pre-mRNA splicing results in nonfunctional TRA and the subsequent default splicing of *dsx* and *fru* pre-mRNA generates Dsx^M^ and Fru^M^ proteins (Boggs *et al.* 1987; Inoue*et al.* 1990; Hoshijima *et al.* 1991; Inoue *et al.* 1992; Ryner *et al.* 1996; Heinrichs *et al.* 1998). Indeed, these gene products sculpt sexually dimorphic traits, reproduction, and behavior (Christiansen *et al.* 2002; Camara *et al.* 2008; Villella and Hall 2008). Previous studies have found that the transcription of sex-biased genes is directly controlled by sex determination transcription factors by binding to the cis-regulatory elements of target gene (Hudry *et al.* 2016; Sawala and Gould 2017; Hudry *et al.* 2019). At present, although huge numbers of sex-biased genes have been identified in insects, studies on the expression profiles of sex-biased genes involved in sex determination are quite limited. Analysis of the differential changes in transcript levels would be very useful for understanding the functional roles of sex-biased genes in sex determination, reproduction, olfaction, immune response, and other progress.

The spotted-wing *Drosophila*, *Drosophila suzukii* (Matsumura), is a global devastating and invasive agricultural pest that causes severe economic loss due to damage to a wide variety of fruit crops such as waxberry, blueberries, strawberries, peaches, cherries, persimmon, and grapes and poses a huge threat to fruit production and security due to its polyphagy, adaptability, and robust fecundity (Lee *et al.* 2011; Walsh *et al.* 2011; Asplen *et al.* 2015; Karageorgi *et al.* 2017; Dweck *et al.* 2021). Whereas other members of Drosophilidae species, such as *D. melanogaster*, lay eggs in overripe fruit, *D. suzukii* lays eggs in ripe fruit, destroying crops through the rot and abscission of fruits. The infestation of *D. suzukii* female to ripe fruits is facilitated by the presence of a large, serrated ovipositor that enables penetrating the surface and deposition of eggs into ripe fruits (Atallah *et al.* 2014; Karageorgi *et al.* 2017; Green *et al.* 2019).

Chemical broad-spectrum insecticides are currently considered to be the most effective tool to control *D. suzukii*. However, their negative impacts on the environment and human health, and the increase in resistance to commonly applied chemical insecticides are strong arguments for the development of novel and environment friendly pest management strategies (Bruck *et al.* 2011; Timmeren and Isaacs 2013; Smirle *et al.* 2017). Recent studies evaluated gene expression profiles in antennae of mated and virgin females, as well as male and female antennae in *D. suzukii* (Crava *et al.* 2019; Ahn *et al.* 2020). However, a comprehensive analysis of sex-specific gene expression in *D. suzukii* male and female adults has not been done yet. So, identification and characterization of sex-biased genes in *D. suzukii* will provide important insights for the development of approaches to produce sterile males and genetic sexing strains based on sex-biased genes in support of sterile insect technique (SIT) applications.

In the current study, we identify candidate genes involved in key biological processes and obtain an overview of sex-biased gene expression profiles by constructing the sex-specific transcriptome in *D. suzukii*. We analyze the differential gene expression between females and males, aiming at elucidating the molecular mechanisms regulating morphological, physiological, and behavioral differences between the sexes and acquiring knowledge about these genes that are potentially involved in sex determination, reproduction, olfaction, and immune response.

## Materials and methods

### Insect rearing

*Drosophila* *suzukii* were fed on an artificial diet consisting of cornmeal, yeast, and agar at Hunan Normal University (Changsha, China). All life stages were cultured at 25°C under 12 h light:12 h dark photoperiod. Newly emerged unmated *D. suzukii* were collected and sorted by sex. Female and male individuals were kept in isolation in standard vials for 3 days.

### RNA extraction and transcriptome sequencing

Three-day-old female and male adults were collected with 3 independently biological replicates, and each replicate contained 10 individuals. Total RNA was extracted from adults of *D. suzukii* using RNAiso Plus reagent (TaKaRa, Dalian, China) according to the manufacturer’s protocol. The degradation and contamination of all RNA samples were monitored on a 1.0% agarose gel, the RNA purity was checked using the NanoPhotometer spectrophotometer (IMPLEN, CA, USA), whereas RNA integrity was assessed using the RNA Nano 6000 Assay Kit of the Bioanalyzer 2100 system (Agilent Technologies, CA, USA). The library construction for Illumina sequencing was conducted with a total amount of 1 μg RNA from female and male adults by using NEBNext Ultra RNA Library Prep Kit for Illumina (NEB, USA) according to the manufacturer’s instructions. Sample sequencing was conducted on an Illumina Novaseq platform using paired-end technology to generate 150-bp reads.

### Transcript sequence analysis

Raw reads of fastq format were firstly processed through in-house perl scripts. In this step, clean reads were generated by removing reads containing adapter, reads containing ploy-N, and low-quality reads from raw datasets. The reads whose low-quality base ratio (base quality ≤5) was more than 20% were removed. At the same time, Q20, Q30, and GC content of the clean data were calculated. All the downstream analyses were based on the clean reads with high quality. The paired-end clean reads were then aligned to the whole-genome sequence (WGS) of *D. suzukii* (https://www.ncbi.nlm.nih.gov/genome/?term=txid28584) with Hisat2 v2.0.5 using the paired information produced by the reference-based aligned reads (Chiu *et al.*, 2013; Paris *et al.*, 2020). The discordant or unpaired alignments were removed. The mapped reads were assembled by StringTie (v1.3.3b) in a reference-based approach. The number of reads mapping to each gene was counted using HTSeq v0.9.1 and the expression level of each gene was further calculated by FPKM (Fragment Per Kilobase of exon model per million mapped reads) based on the length of the gene and read counts mapped to this gene. FPKM considers the effect of sequencing depth and gene length for the reads count simultaneously and is currently the most commonly utilized method for estimating gene expression levels (Arlt *et al.* 2020; Ma *et al.* 2022).

### Differential expression and gene ontology, Kyoto Encyclopedia of Genes and Genomes enrichment analysis

Differential expression analysis of female and male adult libraries was performed using the DESeq2 R package (1.16.1). The resulting *P*-values were adjusted using the Benjamini and Hochberg’s approach for controlling the false discovery rate. Genes with an adjusted *P*-value <0.05 found by DESeq2 were assigned as differentially expressed genes (DEGs). Gene ontology (GO) enrichment analysis of DEGs was implemented by the clusterProfiler R package, in which gene length bias was corrected. GO terms with corrected *P*-value less than 0.05 were considered significantly enriched by DEGs. Kyoto Encyclopedia of Genes and Genomes (KEGG) is a database resource for understanding high-level functions and utilities of the biological system, such as the cell, the organism, and the ecosystem (Kanehisa and Goto 2000). We used clusterProfiler R package to test the statistical enrichment of DEGs genes in KEGG pathways.

### Reverse transcription-PCR

Based on the sequences identified from our transcriptome, *Sxl*, *tra*, *dsx*, and *tra-2* specific primers (Supplementary Table 1) were designed, and PCRs were performed on adult female and male cDNA library templates. For library construction, male and female adults’ total RNAs were extracted separately using RNAiso Plus reagent (TaKaRa, Japan) according to the manufacturer’s protocol. First-strand cDNA was synthesized from 1µg total RNA using PrimeScript Reverse Transcriptase (TaKaRa, Dalian, China) with the oligo (dT) adapter primer. Cycling conditions were denaturation at 94°C for 3 min; followed by 30 cycles of denaturation at 94°C for 30 s, annealing at 55°C for 30 s, and extension at 72°C for 3 min; with a final extension at 72°C for 10 min.

### Quantitative real-time PCR

The transcript expression profiles of candidate genes were investigated using quantitative Real-Time PCR (qRT-PCR). Total RNA was extracted using RNAiso Plus reagent (TaKaRa, Dalian, China) from 10 adults per replicate, with 200 ng for each sample subjected to reverse transcription for mRNA using the PrimeScript RT Master Mix (TaKaRa, Dalian, China). The reverse transcription products were used for qRT-PCR using primers listed in Supplementary Table 1. qRT-PCR was performed using the SYBR Green qRT-PCR mix following the manufacturer’s instructions in a real-time thermal cycler (Bio-Rad, Hercules, CA, USA) using the cycling conditions: 95°C for 10 min, 40 cycles of 95°C for 15 s, 60°C for 30 s, and 72°C for 30 s. Three biological and 3 technical replicates were performed with expression data analyzed by the 2^-△△Ct^ method (Livak and Schmittgen 2001). Dissociation curves were determined for each mRNA to confirm unique amplification. The expression of ribosomal protein 49 (*Rp49*) was used as an internal control to normalize the expression of mRNA.

### Statistical analysis

All qRT-PCR experiments were repeated at least 3 times and analyzed using GraphPad Prism 5.0 (GraphPad Software, San Diego, CA, USA) or Microsoft Excel (Microsoft, Redmond, WA, USA) with results expressed as the mean ± SEM. Data were compared with either a 2-way ANOVA, with subsequent *t*-tests using Bonferroni post-tests for multiple comparisons, or with the Student’s *t*-test. For all tests, differences were considered significant when *P < *0.05.

## Results

### Sequencing and assembly of *D.* female and male adult transcriptomes

Transcriptome libraries of the 3-day-old unmated *D. suzukii* female and male adults were constructed and sequenced in Illumina platform using paired-end sequencing. This generated a total of 349,532,034 125/150 bp-long PE reads with high sequence quality (BioProject accession number: PRJNA668865). After removing low-quality reads, female libraries generated 57, 59, and 56 million clean reads while male libraries generated 54, 62, and 58 million clean reads. Among these clean reads, 41–47 million (75.51–76.64%), were mapped to genes in the WGS of *D. suzukii* (Supplementary Table 2). The percentage of clean reads ranged from 97.88% to 98.46% in female libraries and 97.88% to 98.54% in male libraries (Supplementary Fig. 1). The percentage of reads mapped to the genome exon regions ranged from 74.17% to 79.96% in female libraries and 75.17% to 78.72% in male libraries (Supplementary Fig. 2). The filtered sequence reads from all samples were assembled and produced 16,335 protein-coding genes, 226 pseudogenes, 377 lncRNAs, 350 tRNAs, and 820 unknown biotype genes (Table 1). Gene sequences were annotated using the annotation of *D. suzukii* at NCBI (https://www.ncbi.nlm.nih.gov/genome/annotation_euk/Drosophila_suzukii/102/). A total of 11,909 protein-coding genes (72.9%) were matched to known genes (Supplementary Table 3). The average size of protein-coding genes was 1,926 bp with lengths ranging from 111 to 68,675 bp (Table 1). There were 10,899, 5,228, 2,707, 898, and 108 genes whose length was larger than 1,000, 2,000, 3,000, 5,000, and 10,000 bp, respectively (Supplementary Fig. 3).

**Table 1.** Summary of the *D. suzukii* female and male transcriptomes.

Total reads	349,532,034
Total number of protein-coding genes	16,335
Average transcript length (bp)	1,926
Minimum (bp)	111
Maximum (bp)	68,675
Number of transcripts >1 Kb	10,899
Number of transcripts >2 Kb	5,228
Number of transcripts >3 Kb	2,707
Number of transcripts >5 Kb	898
Number of transcripts >10 Kb	108

### Comparison of gene expression profiles in female and male adults

**Fig. 1.**
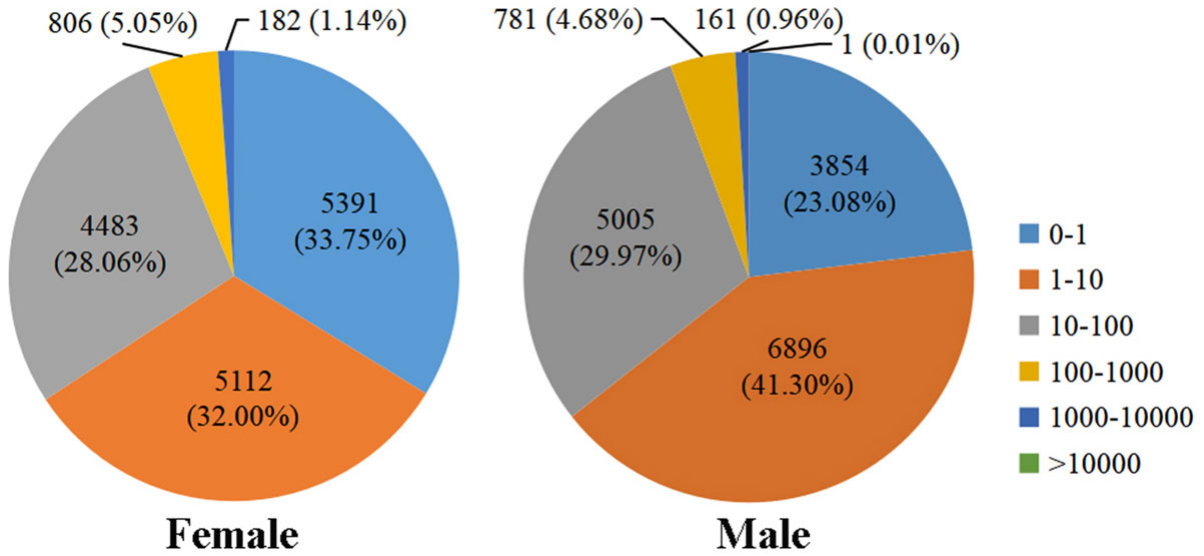
FPKM distribution of the *D. suzukii* female and male adult transcriptomes.

**Fig. 2.**
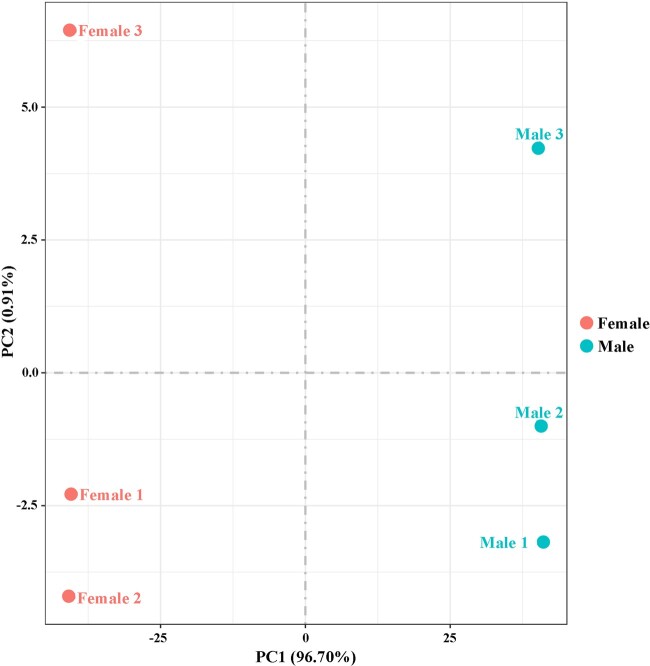
Principal component analysis of the 6 transcriptome samples. Samples are female and male.

**Fig. 3.**
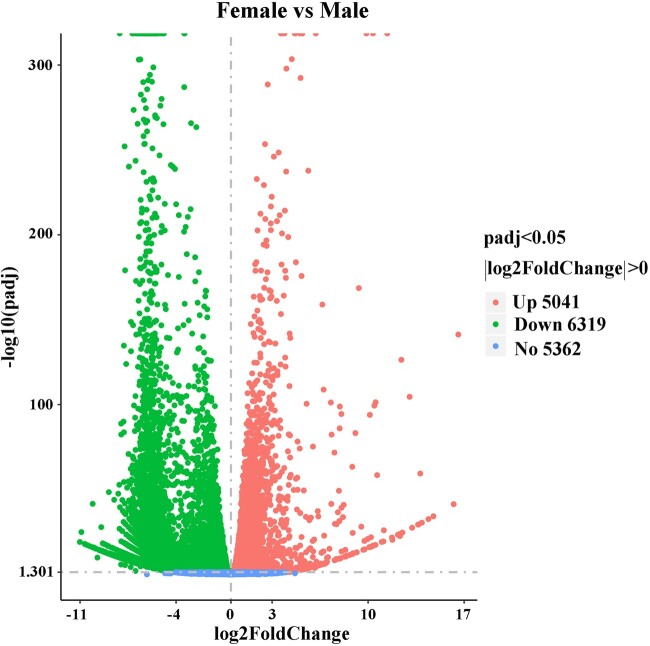
Volcano plot showing DEGs (|log2(FoldChange)| > 0 and *P*adj < 0.05) in female and male adult transcriptomes.

To evaluate the relative expression level of genes in the *D. suzukii* female and male adult transcriptomes, gene read counts were normalized by transforming them into FPKM. We obtained a wide range of expression levels from less than 1 FPKM to approximately 11,182 FPKM (Supplementary Table 3). 65.75% and 64.38% of the genes had low expression level (FPKM 0–10), 28.06% and 29.97% of the genes had moderate expression level (FPKM 10–100), while 6.19% and 5.65% exhibited high expression level (FPKM >100) in the female and male libraries, respectively (Fig. 1). Principal component analysis of all 6 samples showed that both female and male samples clustered together with their respective replicates (Fig. 2). We further identified DEGs between males and females. At a setting of *P* < 0.05, 11,360 DEGs were identified. In total, 6,319 DEGs exhibited relatively higher expression levels in males than females, and 5,041 DEGs showed relatively higher expression levels in females than males (Fig. 3 and Supplementary Table 4). Moreover, we identified that the expression of 584 DEGs was sex specific (sex-specifically expressed genes, SEGs), with 507 SEGs expressed only in male adults, and 77 SEGs expressed only in female adults (Supplementary Table 5).

### Gene function annotation and analysis

**Fig. 4.**
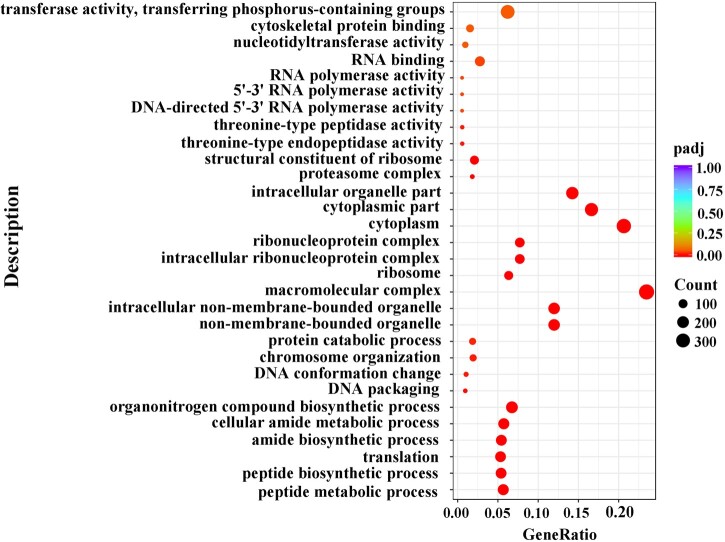
GO significant enrichment analysis for DEGs between female and male transcriptomes of *D. suzukii*.

**Fig. 5.**
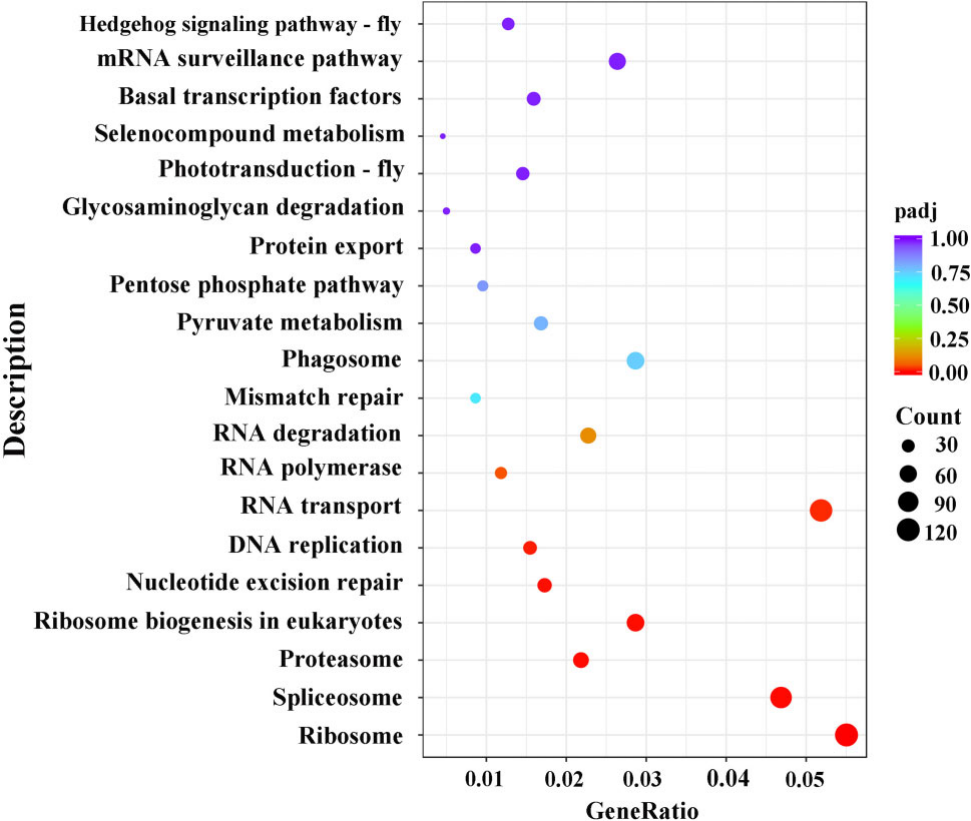
KEGG significant enrichment analysis for DEGs between female and male transcriptomes of *D. suzukii*.

**Fig. 6.**
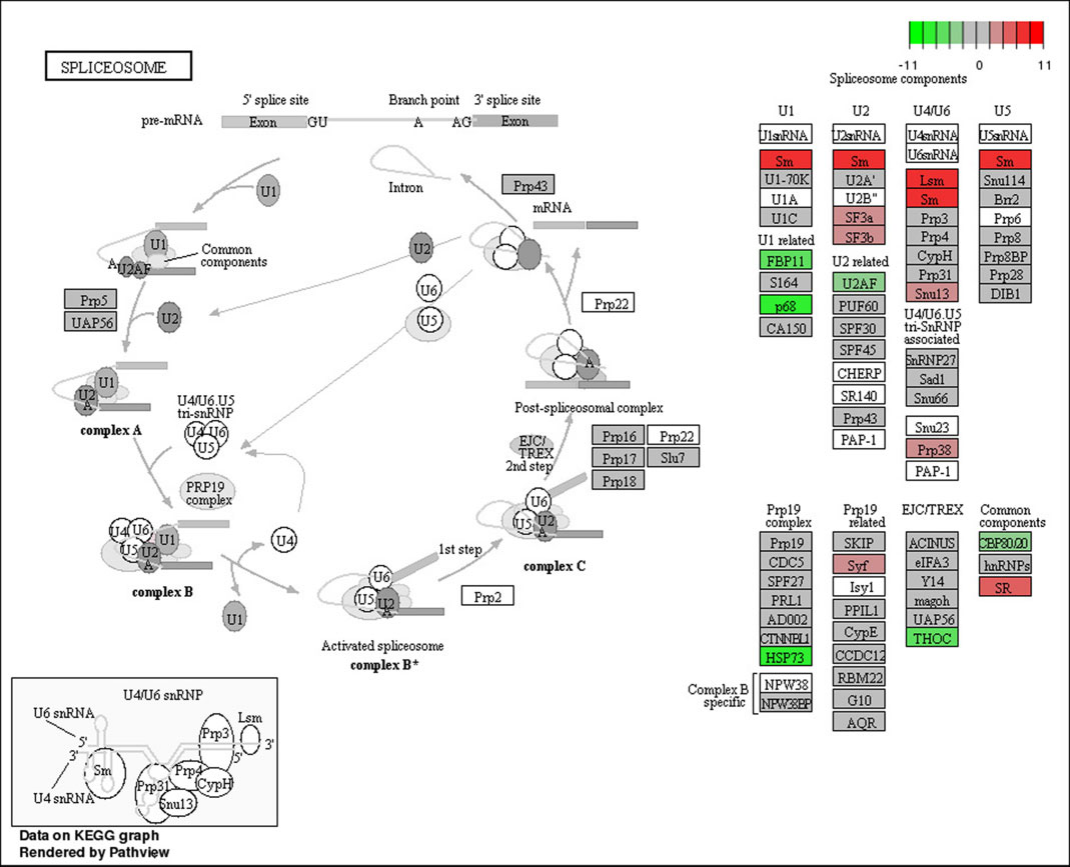
The KEGG pathway of the spliceosome pathway responds to sex-specific splicing between female and male transcriptomes of *D. suzukii*.

We then analyzed the functions of DEGs based on GO classification. In total, 11,360 DEGs were characterized into 3 groups: 2,967, 1,594, and 4,750 DEGs categorized into biological processes, cellular components, and molecular function, respectively (Supplementary Fig. 4 and Supplementary Table 6). The largest representations were in peptide metabolic process and peptide biosynthetic process (biological process), nonmembrane-bounded organelle and intracellular nonmembrane-bounded organelle (cellular component), and structural constituent of ribosome (molecular function). In addition, enrichment comparisons showed that 19 DEGs were involved in sexual reproduction, and 16 DEGs were involved in spermatogenesis, spermatid development, and differentiation in the biological processes (Fig. 4 andSupplementary Fig. 4). Pathway analysis was also conducted using the KEGG annotation system (Fig. 5 and Supplementary Table 7). Of different KEGG annotations, spliceosome pathway was the most enriched in the comparison indicating that sex-specific alternative splicing of sex-biased genes plays a pivotal role in sex determination and differentiation (Fig. 6). Overall, the numbers of skipped exon, mutually exclusive exon, alternative 5′ splice site (A5SS), Alternative 3′ splice site (A3SS), and Retained intron genes which show significant differences between females and males are 251, 54, 41, 74, and 25, respectively (Supplementary Table 8).

### Identification and analysis of sex-biased genes in *D. suzukii*

We identified in total 1,957 female-biased and 4,231 male-biased genes at a threshold of |log_2_(FoldChange)| > 1 and *P*adj < 0.01 (Supplementary Fig. 5 and Supplementary Table 9). The ratio of female-biased and male-biased genes was 10.81% and 23.37%, respectively, in *D. suzukii*, while this ratio for *D. melanogaster* was 3.6% and 13.6% analyzed under the same cutoff. First, consistent with the observation in *D. melanogaster*, many sex-biased genes reported for fertility were found, including *vitellogenin*, *vitellogenin receptor*, genes coding vitelline membrane protein and chorion protein in females, and genes coding accessory gland protein, sperm-specific protein and accessory gland-specific peptide in males. However, genes involved in immunity, metabolism, and chemical sensation were found to be differentially sex-biased between *D. suzukii* and *D. melanogaster*. We identified in *D. suzukii*, a number of female-biased genes in the Toll and IMD immune pathways such as *cecropin C* (*CecC*), *cecropin A2* (*CecA2*), *defensin* (*def*), *diptericin* (*dpt*), *diptericin A* (*dptA*), *pelle* (*pll*), *drosomycin* (*drs*), *metchnikowin* (*mtk*), while only *drs* gene was female-biased and *cecropin A1* (*CecA1*) was male-biased in *D. melanogaster*. For genes involved in metabolism, *Fatty acid synthase* (*FASN*), *lipase 1* (*lip1*), *lipase 3* (*lip3*), *Acyl-CoA synthetase family member 3* (*Acsf3*), *phospholipase A1* (*PlA1*), *glycogen synthase* (*GlyS*), *trehalose transporter 1* (*Tret1*) were male-biased, and *alcohol dehydrogenase* (*Adh*), *Acyl-CoA synthetase family member 4* (*Acsf4*), *Acyl-CoA synthetase short-chain* (*Acss*) were female-biased in *D. suzukii*. However, among the above-mentioned metabolism genes, only *Acyl-CoA synthetase* (*Acs*) was male-biased in *D. melanogaster*. For genes involved in chemical sensation, multitudinous male-biased olfaction genes were identified in *D. melanogaster*, namely, *OBP 56d*, *44a*, *22a*, *50b*, *56g*, *57b*, *56i*, *56f*, *57c*, *51a*, *99c*, and *gustatory receptor 93d* (*GR93d*), whereas only *OBP 19c* was identified as female-biased olfaction gene. In contrast, *D. suzukii* expressed less female-biased genes in this category, with only *odorant-binding protein* (*OBP*) *56a*, *99a*, *99a-like* in females and *odorant receptor* (*OR*) *65 b*, *OBP A5*, and *OBP 56 h* in males being sex-biased. All these results indicate that although the reproduction process requires the similar set of sex-biased genes in both *D. suzukii* and *D. melanogaster*, these 2 *Drosophila* species have opted to diversify sex-biased genes regulating organismal physiology, ranging from immune response, energy metabolism to sensory perception.

### *Drosophila suzukii* sex determination and sex-related reproduction genes

**Fig. 7.**
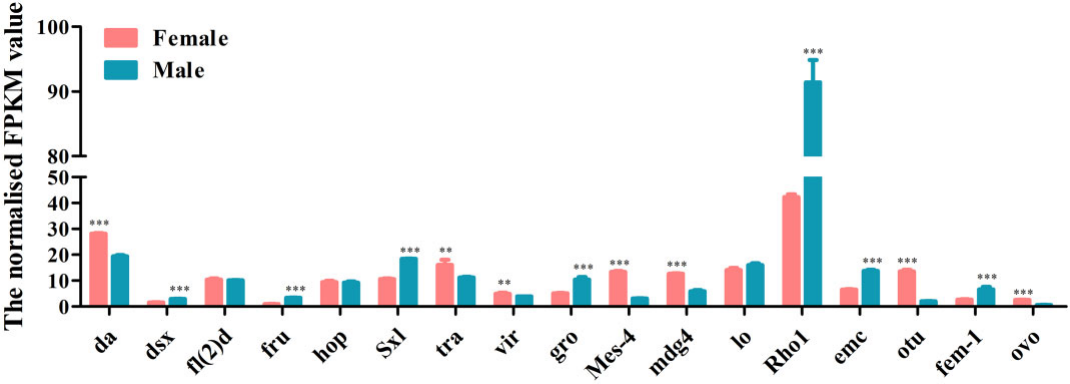
Expression profiles of sex determination transcripts identified in *D. suzukii* female and male transcriptomes. Expression in each sample is reported as the normalized FPKM value. Error bars indicate the SEM of 3 independent biological replicates and asterisks (** and ***) indicate the statistically significant differences (*P* < 0.01 and *P* < 0.001) between female and male adults based on Student’s *t*-test. Sex determination transcripts with greater than 2 FPKM value are shown.

**Fig. 8.**
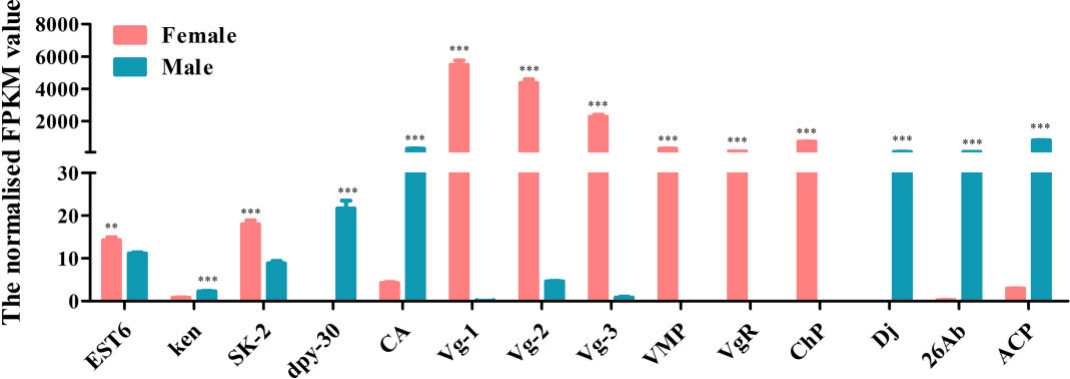
Expression profiles of sex-related reproduction transcripts identified in *D. suzukii* female and male transcriptomes. Expression in each sample is reported as the normalized FPKM value. Error bars indicate the SEM of 3 independent biological replicates and asterisks (** and ***) indicate the statistically significant differences (*P* < 0.01 and *P* < 0.001) between female and male adults based on Student’s *t*-test. Sex-related reproduction transcripts with greater than 2 FPKM value are shown.

**Fig. 9.**
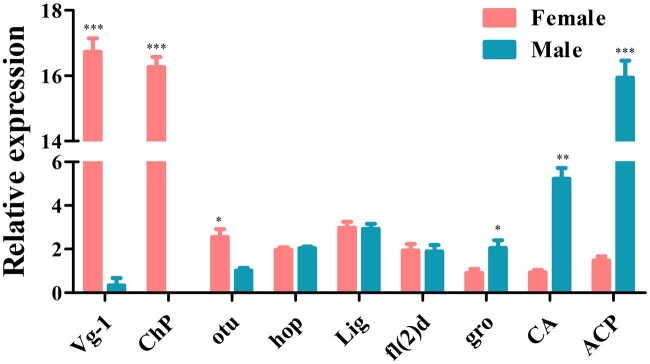
Expression pattern analysis by qRT-PCR in female and male adults. Error bars indicate the SEM of 3 independent biological replicates and asterisks (*, **, and ***) indicate the statistically significant differences (*P* < 0.05, *P* < 0.01, and *P* < 0.001) between female and male adults based on Student’s *t*-test.

A total of 22 genes, including *Sxl*, *tra*, *dsx*, and *fru* which are involved in the sex determination pathway, and *sisterless A* (*sisA*), *scute* (*sc*), *runt*, *deadpan* (*dpn*), and *groucho* (*gro*) which encode X-chromosome-linked signal elements (XSEs), have been identified in *D. suzukii*. We found that 7 of the identified sex determination genes were highly expressed in females and 7 in males while 8 genes showed no difference between the 2 sexes (Fig. 7 and Supplementary Table 10). Although *tra-2* transcript was not found in our transcriptome data, we have isolated a single *tra-2* transcript from both sexes by PCR (Supplementary Fig. 6). Contrary to *tra-2*, we have identified *Sxl*, *tra*, *dsx* genes, which have sex-specific transcripts using Reverse Transcription-PCR (RT-PCR) (Supplementary Fig. 7). *Sxl* gene comprised 1 female and 1 male transcript and *tra* gene comprised 1 female and 2 male transcripts. The male transcripts of *Sxl* and *tra* encode short, nonfunctional proteins because of the stop codons in the male-specific exon while the female transcript encodes a functional protein. *dsx* gene included 1 female and 1 male transcript which encoding a functional female-specific protein and a functional male-specific protein (Supplementary Fig. 6). We further identified a total of 16 sex-related reproduction genes, including *vitellogenin* (*Vg*), *vitellogenin receptor* (*VgR*), vitelline membrane protein (*VMP*), and chorion protein (*ChP*) which are involved in the oogenesis, and sperm-specific protein *Don juan* (*DJ*), accessory gland-specific peptide 26Ab (*26Ab*) and accessory gland protein (*Acp*) which are involved in the spermatogenesis. Eight of them were highly expressed in females, 7 in males and only one showed no difference between them (Fig. 8 and Supplementary Table 10). To confirm the transcriptome data, a group of 9 genes was randomly selected for qRT-PCR. Consistent with our deep sequencing data, qRT-PCR results showed that *Vg-1*, *ChP*, *ovarian tumor* (*otu*) were indeed expressed in a female-biased manner, while the expression of *hopscotch* (*hop*), *lingerer* (*Lig*), *female-lethal(2)d* (*fl(2)d*) had no significant differences between female and male adults. Furthermore, *groucho* (*gro*), *cytosol aminopeptidase* (*CA*), *ACP* showed male-biased expression (Fig. 9).

### *Drosophila suzukii* sex-biased olfaction and immune response genes

**Fig. 10.**
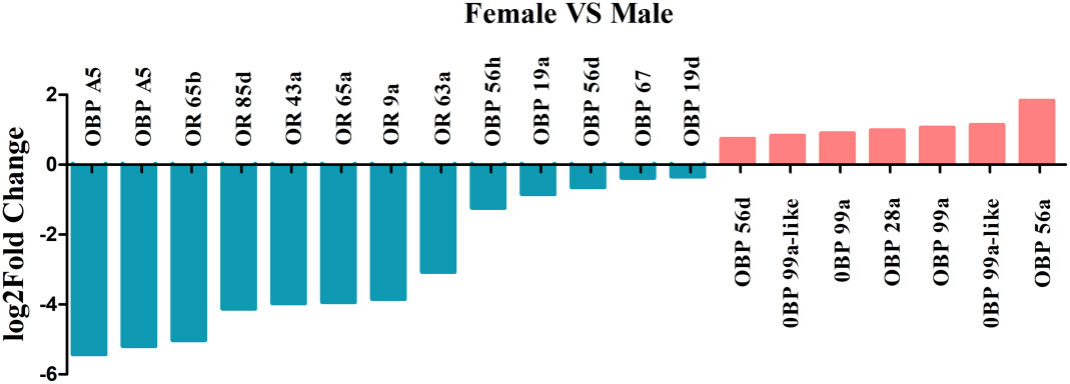
Sex-biased of the olfactory transcripts identified in *D. suzukii* female and male adult transcriptomes.

To evaluate the differential expression of olfactory genes in the *D. suzukii* female and male adult transcriptomes, we identified 123 transcripts encoded by 24 OBPs, 57 ORs, and 3 GRs, and 20 of these transcripts were expressed in a sex-biased manner (Supplementary Table 11). Among them, transcripts encoding 5 OBPs were upregulated in females, while transcripts encoding 6 OBPs and 6 ORs, were upregulated in males. We observed upregulation of OBP 28a, 56a, 56d, 99a, and 99a-like in females, upregulation of OR 9a, 43a, 63a, 65a, 65b, 85d, and OBP A5, 19a, 19d, 56d, 56 h, 67 in males, whereas there was no difference in the expression of GR genes between males and females (Fig. 10 and Supplementary Table 11). Interestingly, OR 43a, 65a, 65b, and 85d were exclusively expressed in males and may play a fundamental role in sex pheromone detection in females (Chauhan *et al.* 2016; Crava *et al.* 2019; Ahn *et al.* 2020). To analyze the difference in sex-specific immunity in *D. suzukii* female and male adult transcriptomes, we identified many genes of innate immune signals (Supplementary Table 3). Among them, 47 transcripts were upregulated in females, while 16 transcripts were upregulated in males. Genes encoding for antimicrobial peptides such as Attacin, Defencin, Cecropin, Diptricin, and immune-induced peptides were among the most differentially upregulated transcripts in females (Supplementary Table 12). Apart from these genes, we also observed upregulation of several other components of the innate immunity pathway in females, including peptidoglycan recognition proteins (PGRPs) (such as PGRP-SB, PGRP-SC, PGRP-LA, and PGRP-LC), gram-negative bacteria-binding protein, Toll, protein toll-like, tyrosine-protein kinase, and NF-kappa-B inhibitor cactus. The upregulation of Toll in the Toll signaling pathway, PGRPs in the Immune deficiency (Imd) signaling pathway and prophenol oxidase in the prophenol oxidase signaling pathway suggest a higher level of basal immune response in *D. suzukii* females.

## Discussion

In this study, we generated female and male transcriptomes of adult *D. suzukii* by next-generation sequencing technology. A total of 11,360 DEGs were identified, among which the expression pattern of 1,957 DEGs were female-biased and 4,231 DEGs were male-biased. Twenty-two sex-determining genes and 16 sex-related reproduction genes were identified, and differential expression profiles of transcripts were validated between female and male transcriptomes. As the first transcriptome analysis of sex-biased genes involved in sex determination, reproduction, olfaction, and immune processes in *D. suzukii*, this dataset could provide a valuable resource necessary for better understanding of the genetic basis of sexual dimorphism in this Drosophilid fruit fly.

Generally, we observed greater amount of genes with male-biased expression relative to female-biased expression in *D. suzukii* (23.37%: 10.81%), reminiscent of features found for *D. melanogaster* (13.6%: 3.6%), *D. simulans* (11.8%: 6.3%), *D. yakuba* (13.5%: 7.8%), *D. ananassae* (7.9%: 4.5%), and *Drosophila* *virilis* (10.8%: 8.6%), and contrasted with *Drosophila* *pseudoobscura* (16.4%: 15.5%) and *Drosophila* *mojavensis* (12.8%: 12.2%; Zhang *et al.* 2007). Previous studies have revealed that male-biased genes show a greater size of bias than female-biased genes (Ranz *et al.* 2003; Ellegren and Parsch 2007; Parsch and Ellegren 2013; Rowe *et al.* 2018) and exhibit more rapid divergence in protein-coding sequence and gene expression level (Zhang *et al.* 2004; Jagadeeshan and Singh 2005; Zhang and Parsch 2005; Proschel *et al.* 2006; Haerty *et al.* 2007; Zhang *et al.* 2007; Baines *et al.* 2008; Meisel 2011; Grath and Parsch 2012; Muller *et al.* 2012). The patterns of sex-biased genes in *D. suzukii* and other *Drosophila* species indicated that male traits might be more evolvable than female traits, possibly endowing males an advantage in sexually antagonistic evolution or potentiating males to avoid resource competition with females (Allen *et al.*, 2018). In addition, we observed that many sex-biased genes participated in reproduction, in line with the observation in *D. melanogaster* (Ellegren and Parsch 2007). Interestingly, genes involved in the immunity, metabolism, and olfaction were differentially sex-biased in *D. suzukii* and *D. melanogaster*. More sex-biased immunity genes in *D. suzukii* than in *D. melanogaster* might reflect a difference in microbial structure and/or load confronted by respective species, or alternatively a distinct basal immune activity (Belmonte *et al.* 2020). Numerous sex-biased genes in metabolism especially lipid metabolism and glycometabolism in *D. suzukii* might be linked to the complex ecological niche and different requirements for the reproduction between sexes (Lee *et al.* 2011; Walsh *et al.* 2011; Dweck *et al.* 2021). However, few sex-biased metabolism genes were identified using transcriptome of whole body samples, despite that metabolism genes can be strongly sex-biased in certain tissues. For example, genes with putative functions in carbohydrate transport and utilization were male-biased in the intestine of *D. melanogaster* (Hudry *et al.* 2019). Furthermore, the different patterns of sex-biased olfaction genes between *D. suzukii* and *D. melanogaster* might underline the distinct oviposition behavior in these 2 species, whereby *D. melanogaster* lay eggs in overripe fruits and *D. suzukii* in ripe fruits (Crava *et al.* 2016; Ramasamy *et al.* 2016; Karageorgi *et al.* 2017; Crava *et al.* 2019). Thus, the transcriptomic studies in *D. suzukii* should enable further understanding of sex-specific physiological processes among different *Drosophila* species.

Sex determination is an essential biological process and involves a cascade of transcription factors that leads to sexual dimorphism in morphology, physiology, and behavior in insects (Sánchez 2008). In *D. melanogaster*, the combination of double XSEs, namely *sisA*, *sc*, *runt, dpn*, and *gro*, act as the primary signal to initiate the sex determination cascade *Sxl* > *tra*/*tra-2 *>* dsx/fru* genes (Cline 1988; Torres and Sánchez 1991; Younger-Shepherd *et al.* 1992; Sánchez *et al.* 1994; Lu *et al.* 2008; Hashiyama *et al.* 2011). The expression of binary switch gene *Sxl* is regulated by the XSEs in a complicated manner in *Drosophila*. The numerator class of primary signal gene *sisA* positively regulates *Sxl*, while the denominator gene *dpn* and maternal gene *gro* negatively regulate *Sxl* expression (Cline 1988; Torres and Sanchez 1991; Younger-Shepherd *et al.* 1992; Penalva and Sánchez 2003; Harrison 2007; Lu *et al.* 2008). In our study, numerous sex-biased genes contributing to sex determination were identified, such as homologs of *sisA*, *sc, runt*, *dpn*, *gro*, *Sxl, tra*, *dsx*, and *fru* but not *tra-2*. Besides that, our transcriptome also uncovered other genes, *fl(2)d* and *virilizer* (*vir*), involved in the auto-regulation of *Sxl* expression (Granadino *et al.* 1990; Niessen *et al.* 2001). Therefore, consistent to previous reports, *Sxl*, *tra*, *dsx*, and *fru* genes play an important role in *D. suzukii* sex determination, by regulating sex-specific alternative splicing (Sánchez 2008; Salz 2011; Li and Scott 2016; Li and Handler 2017). Interestingly, we observed that the spliceosome pathway, alternative splicing events, and the differential expression of sex determination genes were highly enriched in either female or male adults. By experimental verification, we confirmed the sex-specific alternative splicing of *Sxl*, *tra*, and *dsx* genes while *tra-2* gene had the same transcript in males and females. For *A. gambiae* mosquitoes, the CRISPR/Cas9 gene drive targeting of the terminal *dsx* sex determination gene has been used to induce female sterility, resulting in incomplete population suppression (Kyrou *et al.* 2018). In the silkworm *Bombyx mori*, a female-specific embryonic lethal system has been developed by the CRISPR/Cas9 targeting of the *tra-2* gene (Zhang *et al.* 2018). In addition, female-specific lethality strategy can also be achieved by integrating lethal effector gene into the sex-determining genes such as *Sxl*, *tra*, *dsx* which possess the sex-specific alternative splicing for the female-specific expression of lethal effector gene as reported in other economically important pests (Fu *et al.* 2007; Jin *et al.* 2013; Tan *et al.* 2013). For *D. suzukii*, it should be possible to obtain male-only progeny by knocking down or knocking out *tra*, *tra-2*, *dsx* genes and to construct female-specific lethality transgenic strain by targeting *Sxl* gene with CRISPR/Cas9 to make gene drive. Although the sex determination cascade has been extensively studied in various insect species and is found highly conserved, a global picture of sex-biased genes in *D. suzukii* should help to extend our knowledge of sex determination in this pest and may shed light to new target that can be used to make gene drive.

A number of genes associated with reproductive processes were identified in *D. suzukii*. Our data uncovered female-biased genes related to female reproduction and ovarian development, such as *Vg*, *VgR*, *VMP*, *ChP*, *Sphingosine kinase 2* (*Sk2*), and *esterase 6* (*EST6*). In insects, *Vg* gene encodes for the vitellogenin protein, a precursor of lipoproteins and phosphoproteins involved in egg yolk production (Hagedorn and Kunkel 1979) while *VgR* encodes for the specific receptor in *Vg* uptake, so both *Vg* and *VgR* are involved in oocyte maturation and vitellogenesis (Sappington and Raikhel 1998; Tufail and Takeda 2009). Studies on *Vg* and *VgR* genes have demonstrated that *Vg* not only plays an important role in oogenesis but also regulates behavioral and survival traits (Amdam *et al.* 2012), whereas RNA interference targeting *VgR* gene can significantly suppress egg production and delay ovary development (Amdam *et al.* 2012; Lin *et al.* 2013; Lu *et al.* 2015; Zhang *et al.* 2016; Han *et al.* 2017). *VMP* and *ChP* genes are required for normal chorion formation, egg maturation, and oviposition process (Gaino *et al.* 2008; Sdralia *et al.* 2012; Lou *et al.* 2018). *Sk2* and *EST6* are involved in female reproductive behavior in *Drosophila*, and *Sk2* mutant female individuals showed reduced fecundity due to retention of mature eggs in the ovaries while *EST6* can stimulate egg-laying and delay receptivity to remating in females (Richmond *et al.* 1980; Scott 1986; Herr *et al.* 2004). In contrast, male-biased genes are more related to male reproduction and testis development. Examples of such genes include the sperm-specific protein *DJ*, *ACP*, *ken*, *dpy-30*, *Six6*, *CA*, and GATA-binding factor (*GATA*) genes. *DJ* is a nuclear-encoded and germ-cell specifically expressed protein, which likely participates in the final steps of mitochondrial differentiation within the flagellum and plays a key role in spermatogenesis process in *D. melanogaster* (Santel *et al.* 1997, 1998). *ACPs* are generated by the male accessory glands and transferred along with seminal fluid to females during copulation behavior and are essential for male fertility in most insect species (Walker *et al.* 2006; Dottorini *et al.* 2007). *ken* is essential for male fertility and mutations in *ken* gene have resulted in abnormal external genitalia in *Drosophila* (Castrillon *et al.* 1993; Arbouzova *et al.* 2006). *Six* homologs, *six1* and *six4*, are crucial for gonadal development in mouse and a *GATA* homolog, *GATA4*, is a key regulator of testis differentiation and male differentiation in mammalians (Miyamoto *et al.* 2008; Fujimoto *et al.* 2013). In our study, *dpy-30*, *GATA*, and *CA* are highly expressed in males of *D. suzukii*, indicating that these genes may have a role in male development related to fertility and dosage compensation (Hsu and Meyer 1994; Khatun *et al.* 2018). Overall, the identification of sex-related reproduction genes may also contribute to the development of novel genetic control strategies for *D. suzukii* as has been reported for *A. gambiae* (Hammond *et al.* 2016).

Previous comparative transcriptomic analyses have confirmed that sex-biased genes are related to insect olfaction-mediated behaviors, such as locating hosts, mate attraction, mate searching, or selecting oviposition sites to avoid predators (Liu *et al.* 2012; Chauhan *et al.* 2016; Bin *et al.* 2017; Crava *et al.* 2019; Ahn *et al.* 2020; Liu *et al.* 2020; Xu *et al.* 2021). Generally, insect OBPs and chemosensory proteins (CSPs) bind various odorant molecules in the environment and transport them to the ORs or GRs or ionotropic receptors (IRs) at the olfactory sensory neurons to induce the olfactory signal transduction system (Nakagawa *et al.* 2005; Benton *et al.* 2009). This study has identified 24 OBPs, 57 ORs, 3 GRs, but failed to identify the CSPs and IRs in the female and male transcriptomes of *D. suzukii*. The most differentially expressed olfactory genes between sexes were OBPs whereas OBP 28a, 56a, 56d, 99a, 99a-like were female‐biased, and OBP A5, 19a, 19d, 56d, 56h, 67 were male‐biased. The sex-dimorphic expression of the various OBPs suggests different behavioral response to volatiles. Female-biased OBPs were likely being associated with the reception of mating, perception of host volatile signal, and specific egg-laying behavior of *D. suzukii* (Ramasamy *et al.* 2016; Karageorgi *et al.* 2017; Crava *et al.* 2019). Among the 32 OBPs expressed in the antennae of *D. suzukii*, OBP 19a, 19b, 28a, 58c were female‐biased, and OBP 56a, 83ef, 83g, 99c were male‐biased (Ahn *et al.* 2020). OBP 28a was female-biased both in adults and antennae transcriptomes, likely reflecting its role in the gustation and female feeding behavior (Swarup *et al.* 2014). The male-biased expression of OR 9a, 43a, 63a, 65a, 65b, and 85d found in our study may suggest an essential role in detecting sex pheromone of females (Chauhan *et al.* 2016; Crava *et al.* 2019; Ahn *et al.* 2020). The facts that 10 ORs [Or 10a(i), 13a, 43b, 59b, 59c1, 59c2, 67a1, 67a2, 83b, 92a] were found to exhibit female-biased expression in the antennal transcriptome and they also presented increased expression in antennae of mated females, prompted a more detailed analysis of their sex-dimorphic expression profiles of ORs in future (Crava *et al.* 2019; Ahn *et al.* 2020). Concerning the gustatory receptors, we have identified 3 GRs with no sex-biased expression in the female and male adults which is consistent to their expression profile in female and male antennae (Crava *et al.* 2016; Ahn *et al.* 2020). Overall, our finding of several OBPs and ORs with a sex-biased expression in *D. suzukii* indicates that they may play important sex-specific role in pheromone and odor detection, and thus their role warrants further investigation.

The main role of the innate immune system is to defend and protect hosts from all kinds of exogenous pathogens (Kumar *et al.* 2011). The Toll, Imd, Janus kinase, and the Signal Transducer and Activator of Transcription (JAK/STAT), and prophenol-oxidase cascade are 4 different types of innate immune signaling pathways present in insects. The Toll immune and Imd pathway target Gram-positive and Gram-negative bacteria, respectively, while the JAK/STAT pathway and prophenol-oxidase cascade are activated by bacteria/viruses and PGRPs, respectively (Dziarski and Gupta 2006; Stokes *et al.* 2015). Sexual dimorphism in innate immunue response is widespread among insect species (Chauhan *et al.* 2016; Duneau *et al.* 2017; Leech *et al.* 2019; Belmonte *et al.* 2020) and was also reflected in our dataset. We identified several genes of the innate immune pathways including 13 antimicrobial peptides transcripts and 15 PGRPs transcripts in *D. suzukii* female and male transcriptomes. All the 13 antimicrobial peptides transcripts including Attacin, Defencin, Cecropin, and Diptericin were female-biased in our transcriptomes. This finding is in contrast with a previous study suggesting a male-biased expression in *D. suzukii* antennae (Ahn *et al.* 2020). The upregulation of Toll in the Toll pathway, PGRPs in the Imd pathway and prophenol oxidase in the prophenol-oxidase pathway argues for a higher level of basal immune response in *D. suzukii* females. Sex-specific immunity has also been observed in other insect species (Rolff 2001; Innocenti and Morrow 2010; Vincent and Sharp 2014; Chauhan *et al.* 2016). The discrepancy in sex-specific immunity in insects may be associated with the trade-off between immunity and reproduction whereas females can improve their fitness by regulating immunity defense systems appropriately, while in males improved fitness comes by maximizing mating frequency with females. For instance, females of *I. elegans* have a higher baseline immunity level than males possibly to maintain a longer reproductive life and invest more in immune defense as a consequence of the excessive male mating harassment which causes injuries and contributes to the transmission of toxins to females (Chauhan *et al.* 2016). In *Drosophila*, expression of immune genes is sexually dimorphic, both resistance and tolerance to pathogenic infection can be sexually antagonistic (Innocenti and Morrow 2010; Vincent and Sharp 2014).

### Conclusion

In summary, this study reports on the differential gene expression in females and males of *D. suzukii*. Besides providing a comprehensive dataset, we additionally highlight functionally important genes involved in sex determination, reproduction, olfaction, and immune response which may provide insights into the phenotypic and behavioral differences between males and females. This study identified genes which could be potentially useful for the development of genetic sexing strains in support of SIT applications and other genetic control strategies against *D. suzukii*.

## Data availability

All the RNA-sequencing reads have been deposited in the Sequence Read Archive (https://www.ncbi.nlm.nih.gov/sra) with the accession codes (BioProject accession number: PRJNA668865).

Supplementary material is available at *G3* online.

## Supplementary Material

jkac127_Supplemental_Material_Legends

jkac127_Figure_S1

jkac127_Figure_S2

jkac127_Figure_S3

jkac127_Figure_S4

jkac127_Figure_S5

jkac127_Figure_S6

jkac127_Figure_S7

jkac127_Table_S1

jkac127_Table_S2

jkac127_Table_S3

jkac127_Table_S4

jkac127_Table_S5

jkac127_Table_S6

jkac127_Table_S7

jkac127_Table_S8

jkac127_Table_S9

jkac127_Table_S10

jkac127_Table_S11

jkac127_Table_S12
