# Supplementary material for: Transcriptome analysis of sex-biased gene expression in the spotted-wing Drosophila, Drosophila suzukii (Matsumura)
Source: G3 (Bethesda). 2022 May 19;12(8):jkac127. doi: 10.1093/g3journal/jkac127 (PMC9339319; doi:10.1093/g3journal/jkac127)
Supplement: jkac127_Supplemental_Material_Legends [file jkac127_supplemental_material_legends.docx]

**Supplementary material information:**

**Supplementary Table S1** Primers used in our study.

**Supplementary Table S2** Alignment statistics of the *D. suzukii* females and males RNA-Seq analysis.

**Supplementary Table S3** All genes description and FPKM value in *D. suzukii* female and male adult transcriptomes.

**Supplementary Table S4** Differentially expressed genes in pairwise comparison in *D. suzukii* female and male adult transcriptomes.

**Supplementary Table S5** Sex-specific expressed genes in *D. suzukii* female and male adult transcriptomes.

**Supplementary Table S6** GO classification of the differentially expressed genes in pairwise comparison in *D. suzukii* female and male adult transcriptomes. Three main categories, namely biological process (BP), cellular component (CC), and molecular function (MF) were assigned to DEGs.

**Supplementary Table S7** KEGG pathway enrichment analysis for differentially expressed genes in pairwise comparison in *D. suzukii* female and male adult transcriptomes.

**Supplementary Table S8** The numbers of skipped exon (SE), mutually exclusive exon (MXE), alternative 5’ splice site (A5SS), Alternative 3’ splice site (A3SS) and Retained intron (RI) genes which show significant differences between females and males.

**Supplementary Table S9** Sex-biased genes in *D. suzukii* female and male adult transcriptomes.

**Supplementary Table S10** Expression profiles of the sex determination and sex-related reproduction transcripts identified in *D. suzukii* female and male transcriptomes. Expression in each sample is reported as the normalised FPKM value.

**Supplementary Table S11** Sex-biased of the olfactory transcripts identified in *D. suzukii* female and male adult transcriptomes.

**Supplementary Table S12** Sex-biased of the innate immunity signalling pathways transcripts identified in *D. suzukii* female and male adult transcriptomes.

**Supplementary Figure S1** Evaluation of sequence quality for the *D. suzukii* adult females and males.

**Supplementary Figure S2** Percent of reads mapped to the genome regions (exon, intergenic and intron) for the *D. suzukii* adult females and males.

**Supplementary Figure S3** Distribution of protein coding genes lengths in *D. suzukii* female and male adult transcriptomes. The sizes of all protein coding genes were calculated.

**Supplementary Figure S4** Gene ontology classification histogram of differentially expressed unigenes (DEGs) between female and male transcriptomes of *D. suzukii*. Three main categories, namely biological process, cellular component, and molecular function were assigned to DEGs.

**Supplementary Figure S5** Volcano plot showing sex-biased genes (|log2(FoldChange)| > 1 and padj < 0.01) in female and male adult transcriptomes. Female-biased genes shown in red and male-biased genes shown in green.

**Supplementary Figure S6** Expression of *tra-2* gene in *D. suzukii* adult females and males.

**Supplementary Figure S7** Alternative splicing of *Sxl*, *tra* and *dsx* genes in *D. suzukii* adult females and males. *Sxlf*, *traf*, *dsxf*: female specific isoform of *Sxl*, *tra*, *dsx* transcript. *Sxlm*, *tram*, *dsxm*: male specific isoform of *Sxl*, *tra*, *dsx* transcript.
