# Supplementary figures and images for: Transcriptome analysis of sex-biased gene expression in the spotted-wing Drosophila, Drosophila suzukii (Matsumura)
Source: G3 (Bethesda). 2022 May 19;12(8):jkac127. doi: 10.1093/g3journal/jkac127 (PMC9339319; doi:10.1093/g3journal/jkac127)

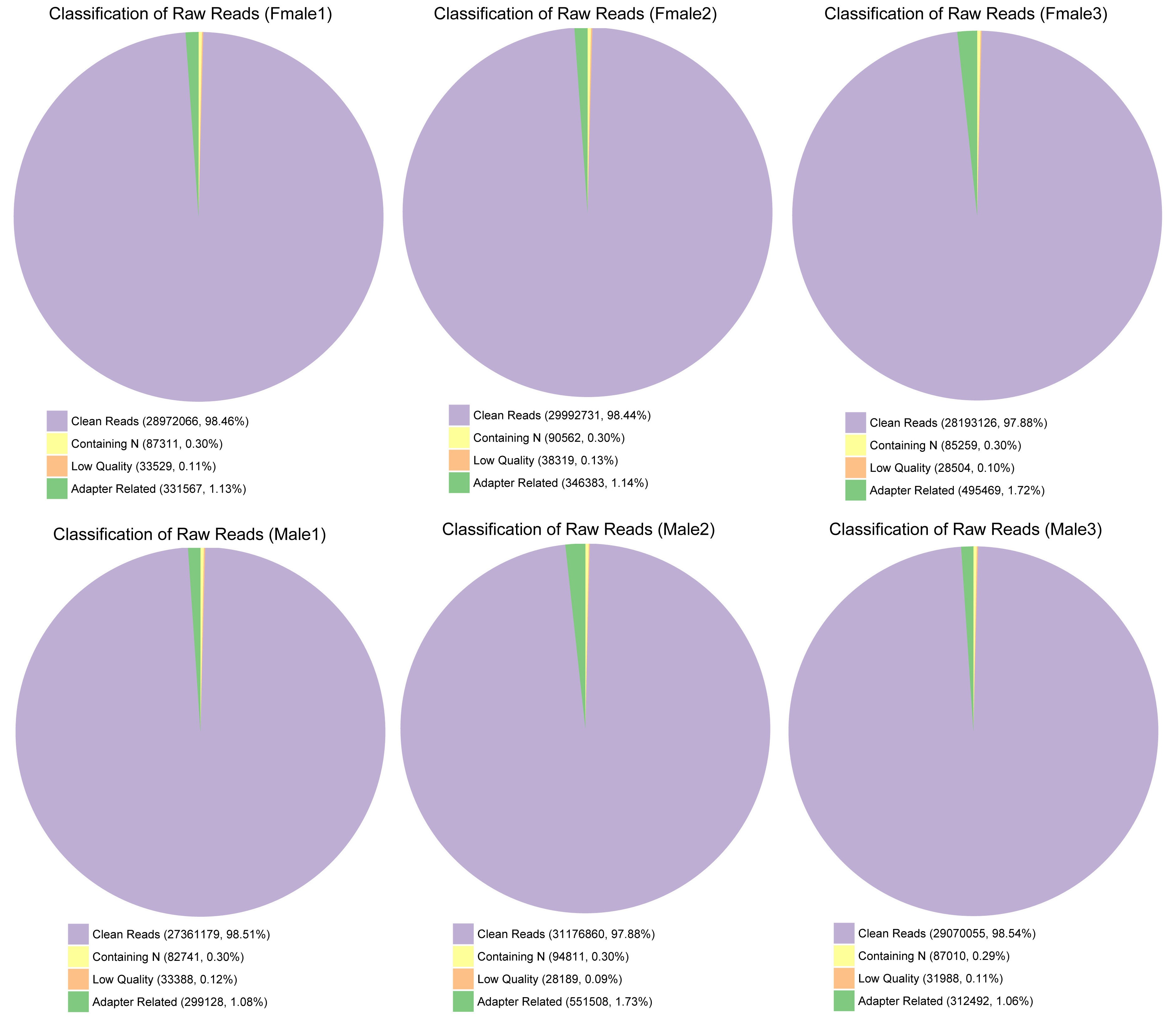

Supplement: jkac127_Figure_S1 [file jkac127_figure_s1.jpeg]

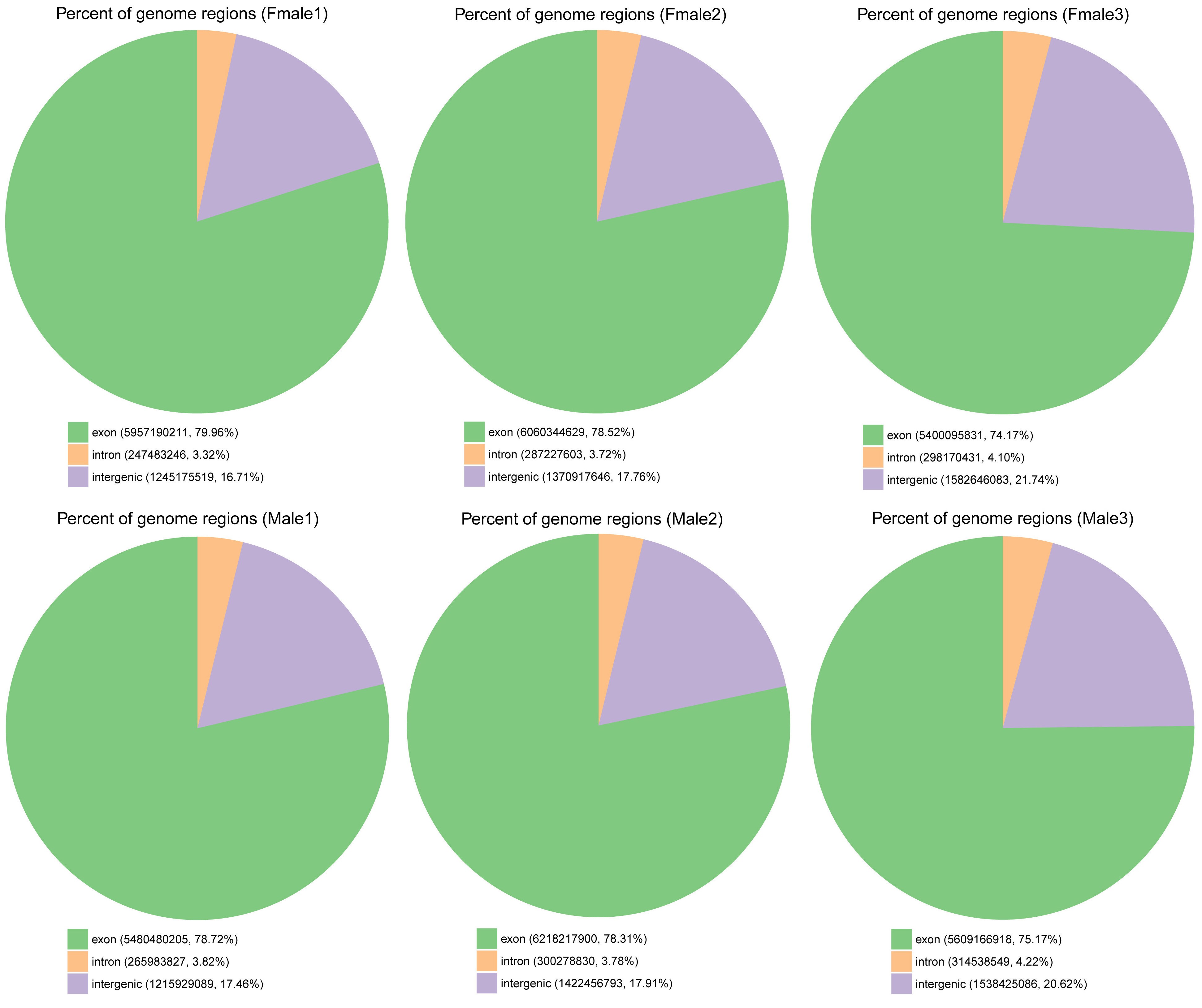

Supplement: jkac127_Figure_S2 [file jkac127_figure_s2.jpeg]

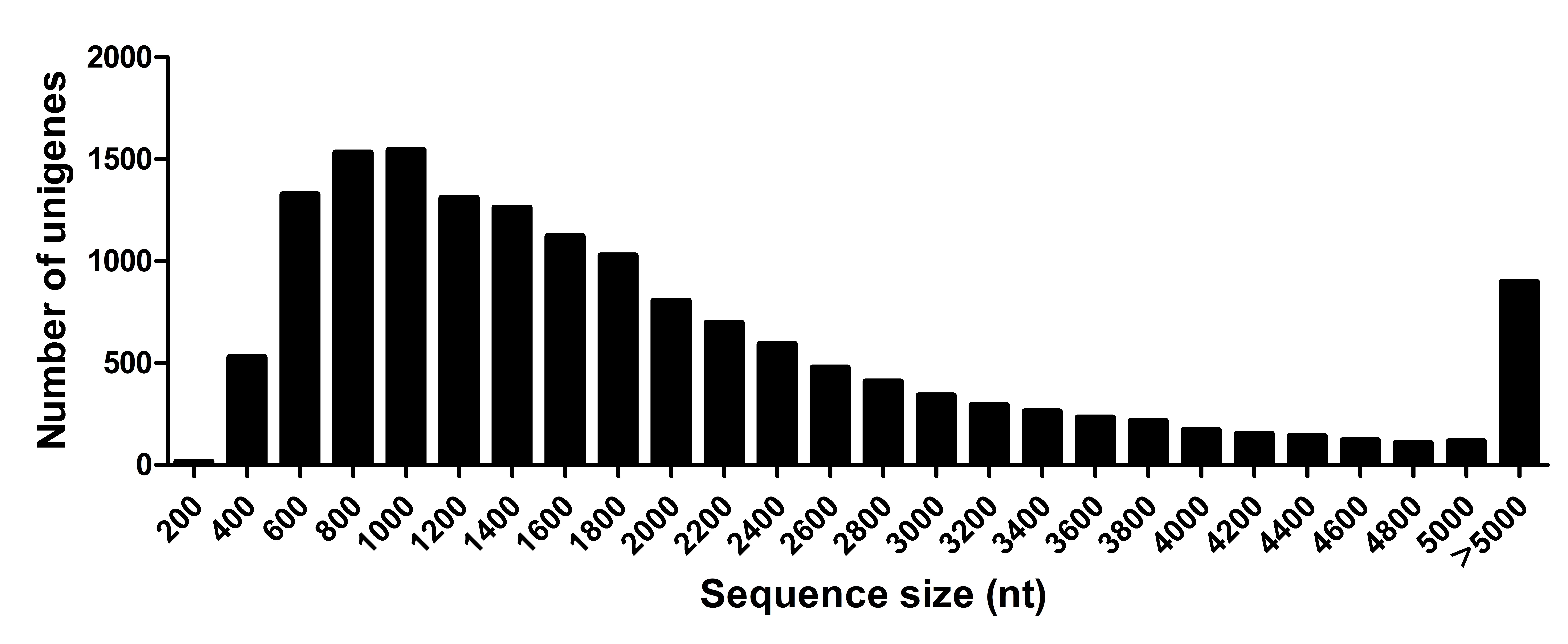

Supplement: jkac127_Figure_S3 [file jkac127_figure_s3.jpeg]

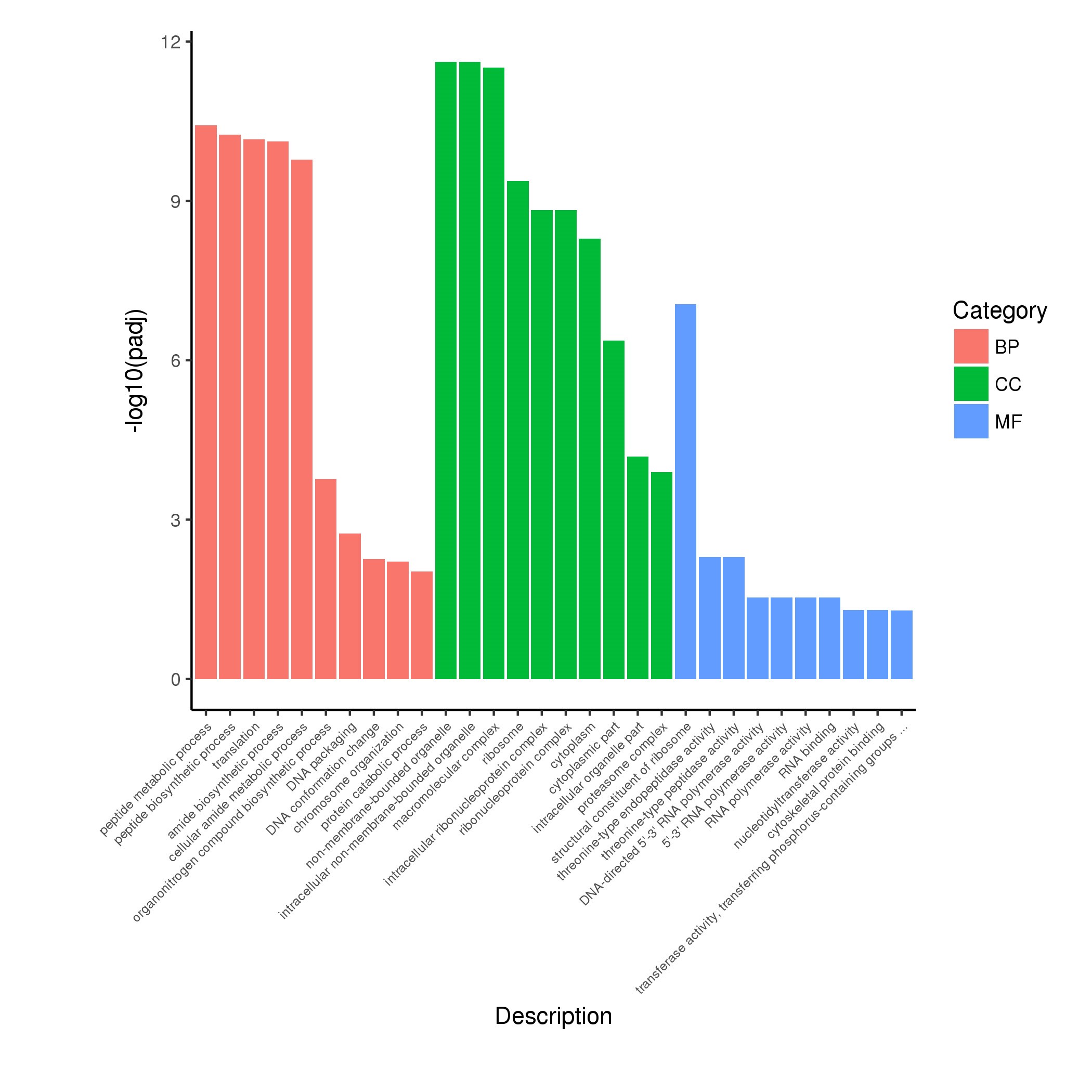

Supplement: jkac127_Figure_S4 [file jkac127_figure_s4.jpeg]

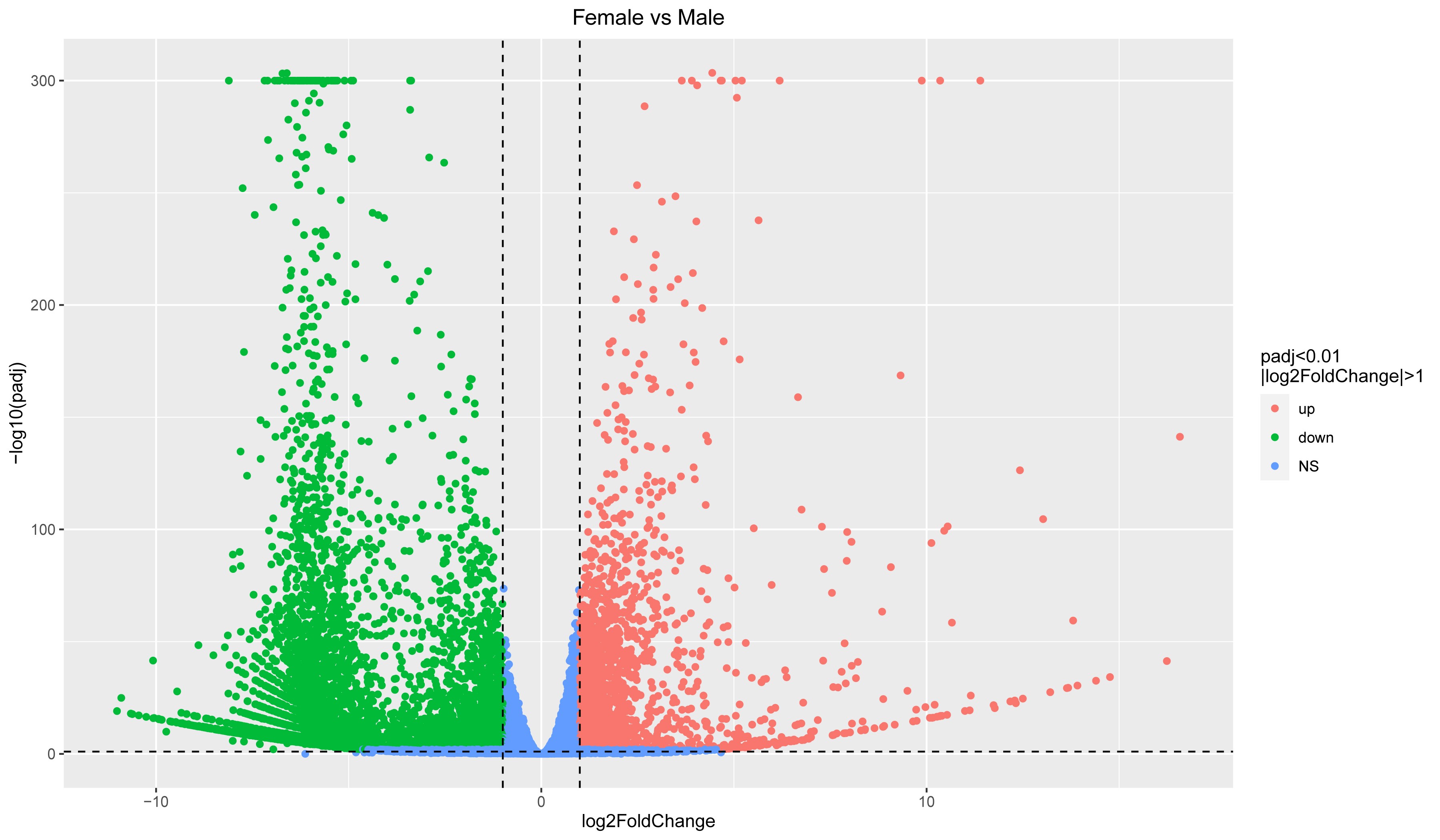

Supplement: jkac127_Figure_S5 [file jkac127_figure_s5.jpeg]

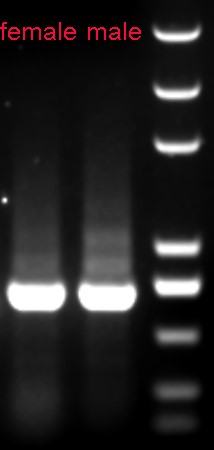

Supplement: jkac127_Figure_S6 [file jkac127_figure_s6.jpeg]

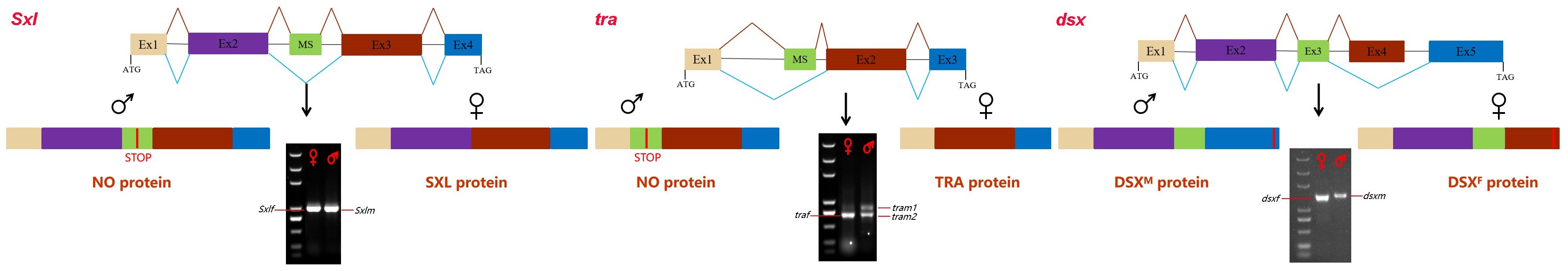

Supplement: jkac127_Figure_S7 [file jkac127_figure_s7.jpeg]
