# Supplementary material for: Transcriptome analysis of sex-biased gene expression in the spotted-wing Drosophila, Drosophila suzukii (Matsumura)
Source: G3 (Bethesda). 2022 May 19;12(8):jkac127. doi: 10.1093/g3journal/jkac127 (PMC9339319; doi:10.1093/g3journal/jkac127)
Supplement: jkac127_Table_S1 [file jkac127_table_s1.docx]

**Table S1** Primers used in our study

| Primer names | Sequence 5’ to 3’ |
| --- | --- |
| RT-PCR primers: | |
| *Sxl F* | TGAACGGCACTAGAATAAAG |
| *Sxl R* | TTGCTGGTTGACAAAATGGT |
| *tra F* | TTGTTAAACGAGTAGTGTCC |
| *tra R* | GTCTAATGTGGTGGTTGC |
| *dsx F* | CAACGGCTCCAGCAATAG |
| *dsx R* | GTGGCTTCATGGATCTGTC |
| *tra-2 F* | GAACCCTTAAAGATGTCCTC |
| *tra-2 R* | CGGCTACGATAATTGTCAG |
| qPCR primers: | |
| *vitellogenin-1* F | CAAGAGCAAGAACACCCT |
| *vitellogenin-1* R | TTGGGATAGAAGTCAACG |
| *chorion protein* F | CCAAGGTTATGGTAGTGC |
| *chorion protein* R | GTACAGTGGAGCCTCGTT |
| *ovarian tumor* F | TACTGCGTCGTCTTTGTG |
| *ovarian tumor* R | ATGGTCGGTACTCGTCTGG |
| *hopscotch* F | ATGTCTATGTGCGGGTCT |
| *hopscotch* R | TGAGGGTGGCTTCTGAGGT |
| *lingerer* F | CTCTGCCTACCTCACATC |
| *lingerer* R | GTCCAGTGTTGCCATAAA |
| *female-lethal(2)d* F | CTGAAGCAGGTTAGCACG |
| *female-lethal(2)d* R | CAGTCTGAATTTGATGGGTT |
| *groucho* F | GGCTCCAGTTCGTCACGTT |
| *groucho* R | GCGGCATCATTTGTTTAGG |
| *cytosol aminopeptidase* F | TAGCGACCCTGAACACTG |
| *cytosol aminopeptidase* R | CTGCTTCCAGATGTAGTG |
| *accessory gland protein* F | ATCCCCGCTTACCAGTAC |
| *accessory gland protein* R | GCCTTTGTCTCAGCTCCA |
| *Rp49* F | CTGCCCACCGGATTCAAG |
| *Rp49* R | CGATCTCGCCGCAGTAAAC |
