# Supplementary material for: Transcriptome analysis of sex-biased gene expression in the spotted-wing Drosophila, Drosophila suzukii (Matsumura)
Source: G3 (Bethesda). 2022 May 19;12(8):jkac127. doi: 10.1093/g3journal/jkac127 (PMC9339319; doi:10.1093/g3journal/jkac127)
Supplement: jkac127_Table_S2 [file jkac127_table_s2.docx]

**Table S2** Alignment statistics of the *D. suzukii* female and male RNA-Seq analysis

| Sample name | Female-1 | Female-2 | Female-3 | Male-1 | Male-2 | Male-3 |
| --- | --- | --- | --- | --- | --- | --- |
| Raw reads | 58,848,946 | 60,935,990 | 57,604,716 | 55,552,872 | 63,702,736 | 59,003,090 |
| Clean reads | 57,944,132 | 59,985,462 | 56,386,252 | 54,722,358 | 62,353,720 | 58,140,110 |
| Clean bases | 8.69 G | 9.0 G | 8.46 G | 8.21 G | 9.35 G | 8.72 G |
| Total mapped | 49,860,464 (86.05%) | 51,656,377 (86.11%) | 48,732,655 (86.43%) | 46,616,723 (85.19%) | 53,168,591 (85.27%) | 49,954,933 (85.92%) |
| Uniquely mapped | 44,405,793 (76.64%) | 45,657,899 (76.11%) | 42,575,889 (75.51%) | 41,748,304 (76.29%) | 47,608,089 (76.35%) | 44,341,835 (76.27%) |
| Multiple mapped | 5,454,671 (9.41%) | 5,998,478 (10.0%) | 6,156,766 (10.92%) | 4,868,419 (8.9%) | 5,560,502 (8.92%) | 5,613,098 (9.65%) |
| Error rate (%) | 0.03 | 0.03 | 0.03 | 0.03 | 0.02 | 0.03 |
| Q 20 (%) | 97.88 | 97.98 | 97.84 | 97.82 | 98.07 | 97.95 |
| Q 30 (%) | 93.89 | 94.14 | 93.73 | 93.74 | 94.31 | 94.01 |
| GC content (%) | 50.1 | 49.63 | 48.33 | 49.11 | 48.97 | 48.04 |
