# Supplementary material for: Transcriptome analysis of sex-biased gene expression in the spotted-wing Drosophila, Drosophila suzukii (Matsumura)
Source: G3 (Bethesda). 2022 May 19;12(8):jkac127. doi: 10.1093/g3journal/jkac127 (PMC9339319; doi:10.1093/g3journal/jkac127)
Supplement: jkac127_Table_S10 [file jkac127_table_s10.docx]

**Table S9** Expression profiles of the sex determination and sex-related reproduction transcripts identified in *D. suzukii* female and male transcriptomes. Expression in each sample is reported as the normalised FPKM value.

| Gene name | Gene ID | Gene length (bp) | Female-1 | Female-2 | Female-3 | Male-1 | Male-2 | Male-3 | Fmale vs Male log2Fold Change |
| --- | --- | --- | --- | --- | --- | --- | --- | --- | --- |
| *daughterless** | 108021200 | 3553 | 28.25 | 27.74 | 28.53 | 19.25 | 18.80 | 20.31 | 0.53 |
| *deadpan* | 108019669 | 1764 | 0.06 | 0.24 | 0.10 | 0.06 | 0.03 | 0.13 | 0.93 |
| *doublesex** | 108011781 | 2699 | 1.45 | 1.78 | 1.47 | 3.03 | 2.84 | 2.94 | -0.90 |
| *female-lethal(2)d* | 108019872 | 3772 | 10.50 | 9.63 | 11.14 | 10.01 | 10.00 | 10.36 | 0.04 |
| *fruitless** | 108020278 | 2965 | 0.70 | 1.08 | 1.01 | 3.37 | 3.51 | 3.26 | -1.86 |
| *hopscotch* | 108016885 | 5100 | 9.61 | 10.30 | 8.31 | 10.12 | 8.75 | 8.83 | 0.03 |
| *scute* | 108016700 | 1295 | 0.08 | 0.16 | 0.05 | 0 | 0 | 0 | 3.71 |
| *sisterless A* | 108004645 | 657 | 0 | 0 | 0 | 0 | 0 | 0 | NA |
| *Sex-lethal** | 108016581 | 1848 | 10.85 | 9.99 | 10.81 | 18.51 | 18.51 | 18.24 | -0.81 |
| *transformer** | 108005620 | 721 | 17.83 | 18.21 | 12.29 | 11.73 | 10.83 | 11.15 | 0.52 |
| *virilizer** | 108009678 | 5680 | 5.43 | 5.22 | 4.00 | 4.03 | 3.83 | 3.82 | 0.33 |
| *groucho** | 108015824 | 1395 | 5.43 | 4.95 | 4.95 | 12.47 | 9.27 | 9.44 | -1.02 |
| *Mes-4** | 108017494 | 4619 | 13.83 | 13.63 | 12.64 | 2.84 | 3.16 | 3.30 | 2.11 |
| *mdg4** | 108007149 | 1929 | 12.45 | 13.07 | 12.38 | 5.32 | 5.82 | 6.65 | 1.09 |
| *longitudinals lacking* | 108006185 | 1689 | 13.80 | 12.68 | 15.70 | 16.61 | 14.73 | 16.75 | -0.19 |
| *Rho1** | 108009598 | 1670 | 44.23 | 40.54 | 42.22 | 85.33 | 91.57 | 97.29 | -1.11 |
| *extra macrochaetae** | 108013509 | 1954 | 6.83 | 6.31 | 6.44 | 13.96 | 14.33 | 12.94 | -1.07 |
| *ovarian tumor** | 108004763 | 3260 | 14.34 | 14.01 | 12.26 | 2.19 | 1.93 | 1.90 | 2.76 |
| *runt* | 108014951 | 1729 | 0.03 | 0.06 | 0 | 0.03 | 0.08 | 0.10 | -1.17 |
| *feminization-1** | 108020775 | 2461 | 2.43 | 2.46 | 3.08 | 6.27 | 5.12 | 8.55 | -1.32 |
| *ovo** | 108016913 | 6770 | 2.25 | 2.71 | 2.62 | 0.68 | 0.58 | 0.58 | 2.04 |
| *doublesex- and mab-3-related transcription factor 2* | 108005351 | 1086 | 0.05 | 0 | 0.11 | 0 | 0.09 | 0 | 0.79 |
| *esterase 6** | 108012386 | 1711 | 13.05 | 14.24 | 15.50 | 11.71 | 10.98 | 10.80 | 0.35 |
| *ken** | 108021721 | 3905 | 0.59 | 0.96 | 0.78 | 2.38 | 2.39 | 2.12 | -1.56 |
| *sphingosine kinase 2** | 108007111 | 2469 | 18.55 | 16.23 | 19.18 | 9.49 | 9.23 | 7.80 | 1.02 |
| *dpy-30** | 108011324 | 481 | 0 | 0 | 0 | 25.06 | 18.71 | 21.401 | -6.85 |
| *Six6* | 108012588 | 1421 | 0.42 | 0.48 | 0.47 | 0.15 | 0.14 | 0.35 | 1.10 |
| *GATA-binding factor** | 108018471 | 1765 | 0.03 | 0 | 0 | 0.23 | 0.19 | 0.06 | -3.89 |
| *cytosol aminopeptidase** | 108019638 | 1803 | 4.47 | 4.42 | 3.80 | 317.77 | 302.63 | 298.11 | -6.17 |
| *vitellogenin-1** | 108014989 | 1575 | 5507.44 | 5941.48 | 5001.58 | 0.07 | 0 | 0.11 | 16.58 |
| *vitellogenin-2** | 108015181 | 1558 | 4492.69 | 4673.02 | 3910.83 | 4.69 | 4.48 | 4.76 | 9.88 |
| *vitellogenin-3** | 108013677 | 1398 | 2318.71 | 2471.26 | 2011.49 | 0.51 | 1.19 | 0.80 | 11.40 |
| *vitelline membrane protein** | 108014282 | 687 | 307.24 | 329.52 | 283.77 | 0 | 0 | 0 | 14.40 |
| *vitellogenin receptor** | 108006561 | 6121 | 129.90 | 131.45 | 119.17 | 0.04 | 0.02 | 0.01 | 12.42 |
| *chorion protein** | 108004584 | 1042 | 748.74 | 749.732 | 673.61 | 0 | 0 | 0 | 16.24 |
| *sperm-specific protein Don juan** | 108017133 | 1175 | 0 | 0 | 0 | 110.25 | 111.37 | 100.89 | -9.16 |
| *accessory gland-specific peptide 26Ab** | 108008575 | 539 | 0.40 | 0.20 | 0 | 99.197 | 102.21 | 95.58 | -8.90 |
| *accessory gland protein** | 108018864 | 2535 | 2.90 | 3.06 | 2.82 | 778.14 | 820.50 | 830.86 | -8.11 |

*** showes significant difference between female and male adult transcriptomes.
